# Dissection of the Genetic Basis of Genotype by Environment Interactions for Morphological Traits and Protein Content in Winter Wheat Panel Grown in Morocco and Spain

**DOI:** 10.3390/plants13111477

**Published:** 2024-05-27

**Authors:** Adil El Baouchi, Mohammed Ibriz, Susanne Dreisigacker, Marta S. Lopes, Miguel Sanchez-Garcia

**Affiliations:** 1 International Center for Agricultural Research in the Dry Areas (ICARDA), Rabat 10100, Morocco; 2 Plant, Animal, and Agro-Industry Production Laboratory, Faculty of Sciences, Ibn Tofail University, Kenitra BP. 242, Kenitra 14000, Morocco; m_ibriz@yahoo.fr; 3 The International Maize and Wheat Improvement Center (CIMMYT), Texcoco 56237, Mexico; s.dreisigacker@cgiar.org; 4 The International Maize and Wheat Improvement Center (CIMMYT), Ankara 3906511, Turkey; marta.dasilva@irta.cat; 5 Sustainable Field Crops Institute for Food and Agricultural Research and Technology (IRTA), 251981 Lleida, Spain

**Keywords:** winter wheat panel, genotype by environment interaction, sowing dates, climatic variables, quality, morphological traits, genome-wide association study

## Abstract

To fulfill the growing demand for wheat consumption, it is important to focus on enhancement breeding strategies targeting key parameters such as yield, thousand kernel weight (TKW), quality characteristics including morphological traits, and protein content. These elements are key to the ongoing and future objectives of wheat breeding programs. Prioritizing these factors will effectively help meet the rising demand for wheat, especially given the challenges posed by unpredictable weather patterns. This study evaluated the morphological traits and protein content of 249 winter wheat varieties and advanced lines grown in eleven different environments in Morocco and Spain incorporating three varied sowing dates. The results showed considerable variability in morphological traits and protein content. Significant correlations were observed among various grain traits, with most grain morphological parameters exhibiting negative correlations with protein content. Differences across environments (*p* ≤ 0.01) in all traits, genotypes, and genotype by environment interaction were significant. A factorial regression analysis revealed significant impacts of environmental conditions on all grain morphological parameters, protein content, and TKW during the three growth stages. The study identified several high-performing and stable genotypes across diverse environments, providing valuable insights for wheat breeding programs such as genotypes 129, 234, 241, and 243. Genome-Wide Association Studies pinpointed 603 significant markers across 11 environments, spread across chromosomes. Among these, 400 markers were linked with at least two traits or observed in at least two different environments. Moreover, twelve marker-trait associations were detected that surpassed the Bonferroni correction threshold. These findings highlight the importance of targeted breeding efforts to enhance wheat quality and adaptability to different environmental conditions.

## 1. Introduction

Bread wheat (*Triticum aestivum* L.) is an important widely grown cereal crop grown with an annual production of 760 M tons [1], occupying the biggest land area under cultivation of one crop in the 21st century with more than 219 million ha planted annually. Wheat end-use products play an essential role in human nutrition contributing to up to 20% of the daily intake of proteins and 21% of food calories. By 2050, the global population is expected to reach 9 billion people on Earth. The production and material to feed this number are not set up today, which will demand approximately a 70% increase in worldwide wheat production [2,3,4]. Wheat grain morphology traits are all grain parameters related to the physical characteristics of the grain. The size, color, and shape of the grain, as well as the ratio of grain width by grain length, can vary depending on the genotype and environmental conditions [5,6] and, therefore, can affect the nutritional content, processing quality, and market value. Grain morphology has been linked to thousand kernel weight (TKW), as well as flour yield and end-use quality [7]. In addition, grain length, width, and TKW influence directly test weight which determines flour quality grade and therefore impacts flour market price for millers [6]. Wheat yield is determined by several complex agronomic traits, such as plant height, earliness, vernalization and photoperiod response, resistance to abiotic stresses, etc. However, grain characteristics also play a major role in increasing yield [8], and are correlated with yield due to their stability and effect on grain weight [9,10,11]. Grain size and weight are considered as main components influencing wheat yield. Longer and wider grains do not only directly influence yield but also positively impact seeding vigor and early development, which in turn promote and stabilize yield potential [12]. However, it is important to consider the potential implications for seed cost. Larger seeds typically require more resources and may result in higher seed costs for farmers. All these morphological grain characteristics as well as TKW are traits highly influenced by the environment [13]. Usually, large grains contain more starch and nutrients than small grains, which leads to higher yield and more flour. To optimize yield and nutritional quality, scientists must consider these factors related to grain morphology by selecting genotypes with desirable shape characteristics for each specific environment to maximize yield, quality, and profitability. In wheat production, yield is influenced by several components, including the number of grains per plant, grain size, and grain weight [14]. For example, it could be advantageous to select genotypes with larger grains in an irrigated environment where water availability is more constant. These characteristics often correlate with higher yield potential and can contribute to better water and nutrient utilization, ultimately enhancing profitability. Conversely, in a rainfed environment where water availability fluctuates smaller genotypes may be more suitable due to their greater drought tolerance and higher water use efficiency, supporting yield stability and profitability. While cultivars with larger grains are not necessarily more drought tolerant, they may exhibit characteristics such as deeper root systems or greater water use efficiency, which can contribute to drought resilience [15]. Therefore, when considering drought tolerance, it is essential to evaluate multiple characteristics beyond grain size. Several environmental factors affect wheat productivity and quality; sowing dates emerge as the key determinant factor. The optimal sowing date in regions like Morocco and Spain usually starts in the second half of November. However, delaying or advancing sowing dates may impact significantly wheat growth, yield, and quality [16]. Early sowing dates may coincide with high-temperature stress during the initial developmental stages, which reduces germination rate and therefore reduces plant population and yield. Late sowing dates may coincide with terminal heat during the flowering and/or grain-filling period, which affect significantly the grain characteristics and therefore yield and quality [17,18,19,20]. Genotype by environment interaction (GEI) is a common phenomenon in all multi-environment trials. They represent a major challenge for breeders who want to develop and advance materials throughout generations that are more adapted to several environmental conditions. The statistical analysis of GEI is a widely used method to identify the optimum value of the targeted trait in several environments [21]. Understanding the GEI effect on grain morphology is therefore an important consideration in bread wheat breeding programs, as it affects not only the agronomic parameters but also quality parameters [22]. The objectives of this study were (1) to study the morphological parameters, including grain shape and size, and protein content of the Winter Wheat Association Genetics Initiative (WWAGI) panel under eleven environments (location × year × sowing date combinations) using multi-environment trial data and identify ideal genotypes with high performance and wide adaptability, (2) to disseminate the effect and importance of the climatic variables on each trait, (3) to identify molecular markers associated with morphological grain traits and protein content.

## 2. Results

### 2.1. Characterization of Environmental Field Trials

**Figure 1:**
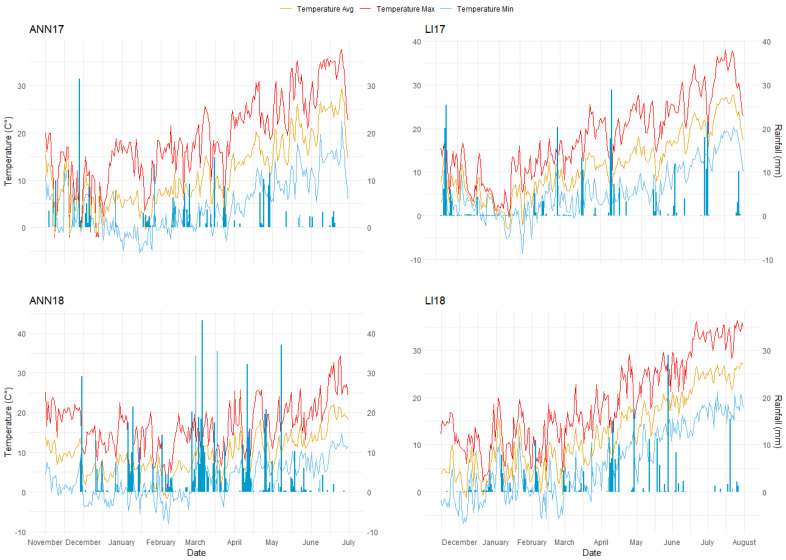
Precipitation (mm), average, maximum, and minimum temperatures (°C) registered at Annoceur (ANN) and Leida (LI) stations during the cropping seasons of 2016–2017 and 2017–2018, categorized for early planting (EP), medium planting (MP), and late planting (LP).

A total of two experimental field stations located in two countries, three planting dates, and two years were used in this study, to characterize 249 modern winter wheat genotypes. Temperature and precipitation varied significantly among the 11 environments used. Weather data of all trials conducted in Morocco and Spain are shown in Table 1 and Figure 1. 

In the early planting (E) treatment, ANN17 showed the highest precipitation with 1060 mm from planting to harvest, followed by ANN18 with 713 mm, LI17 with 295 mm, and LI18 with 263 mm. For the medium planting (M) treatment, ANN18 showed the highest water input with 628 mm, followed by 260 mm for LI18 and 231 mm for LI17, while for ANN17 the precipitation dropped dramatically to 184 mm as compared to the early planting. In the late planting (L), the highest amount was observed in ANN18 with 494 mm, followed by LI18 with 237 mm, LI17 with 223 mm, and ANN17 with 151 mm (Table 1). Overall, these trials could be identified as moderately cold winter with a maximum temperature not exceeding 21.9 °C and 8.2 °C for minimum temperature, gradually higher temperatures, and a reduction in rainfall from early planting toward late planting. Across all environments including locations, years, and sowing dates, the flowering of all entries occurred when average temperature among the stages increased by 1 °C from the period before heading (BH) to grain setting (GS). Additionally, a subsequent temperature rise of 3 °C was detected from grain setting to grain filling (GF) (Figure 1).

### 2.2. Grain Morphological Traits and Protein Content of the WWAGI Genotypes in Multiple Environments

The highest value for grain area was observed in LI17 during the early planting with 17.28 mm^2^. The same pattern was noticed for length, perimeter, TKW, and width with 6.76 mm, 20.69 mm, 44.55 g, and 3.265 mm, respectively, while the lowest value was detected in the late planting with 13.77 mm^2^, 18.63 mm, 30.23 g, and 2.84 mm for area, perimeter, TKW, and width, respectively, in ANN18L. In addition, the result revealed that all traits showed a significant decrease from the first date to the third date, except for grain protein content which was higher on the third date than on the first and second dates (Table 2). Considering all traits, the highest heritability was observed for grain length with a mean *H*^2^ of 0.81 among all environments, while protein content presented the lowest value of heritability with a mean *H*^2^ of 0.43 (Table S1).

The combined analysis of variance showed significant (*p* < 0.001) variation among genotypes, environments, and genotype by environment interaction (GEI) for all the traits as shown by “genotype”, “environment”, and “GE interaction” effects in Table 3, except for color. The GE interaction effect was the main source of variation of almost all traits and contributed up to 60% of the total sum of squares for protein, while for circularity, roundness, and volume, the environment was the main source of variation and contributed up to 53% of total variation. For genotype, the highest value was noticed for color with 40% of the total variation, while the lowest value was detected for protein with only 6%, indicating the highest impact of GE interaction on protein content. Moreover, grain width exhibited a comparatively higher percentage relative to the total sum of squares compared to grain length. This highlights greater responsiveness of grain width to environmental conditions, while the lower influence of GE on grain length suggests a more constitutive and stable genetic control over this particular trait.

### 2.3. Relationship and Correlation between Studied Traits

**Figure 2:**
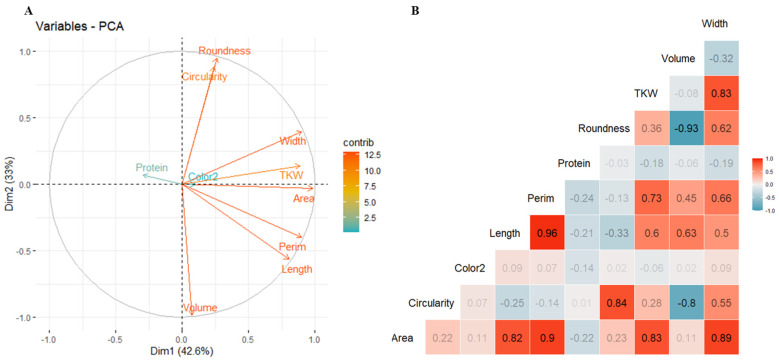
(**A**) Principal component analysis (PCA) biplot of the first two axes for the WWAGI panel based on all traits. The length of the vectors indicates the influence of these traits on the overall dataset. (**B**) Pearson matrix for the analyzed traits; positive correlations are displayed in red and negative in blue.

The evaluation of the mutual dependence between traits is shown in Figure 2. Across all environments, we observed that correlations between all grain traits were highly significant. The first and second axes of the principal component analysis (PCA) of traits explain 76% of the total variation (Figure 2A). Most grain morphological parameters were negatively correlated with protein content suggesting that longer, wider, and heavier grains exhibit a lower percentage of protein.

**Figure 3:**
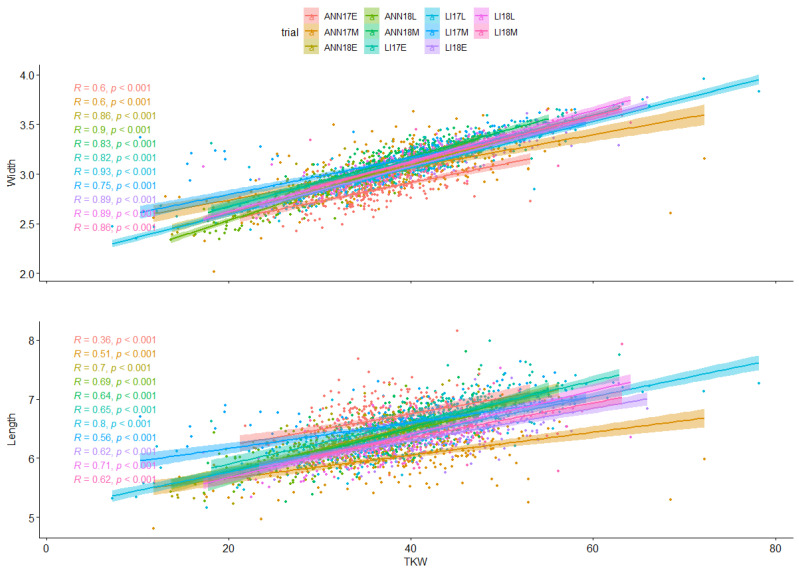
Linear Regression between the three main morphological parameters TKW, width, and length across all environments.

The highest correlation was detected between grain length and perimeter with an r = 0.96, *p* < 0.01 followed by r = 0.93, *p* < 0.01 between grain roundness and volume. However, a distinct pattern was detected when considering the relationship between protein content and most of the grain morphological traits exhibiting a negative correlation. Furthermore, the genetic regression analyses between grain length and width as compared to TKW were calculated per environment and expressed graphically using linear regression (Figure 2 and Table S2). The results showed that all environments were significant to highly significant for both traits. The relationships between grain width and TKW were highly significant across all environments, ranging from r = 0.64 to 0.93, suggesting a robust association between grain width and TKW, irrespective of the environmental conditions. The strongest correlation was detected in the late planting (environment with the highest drought conditions), registering an R^2^ of 0.93 for LI17L, followed by 0.9 for ANN18L, indicating that grain width could be considered as a proxy for grain shriveling, while for the relationships between grain length and TKW, the association was not high but remained significant and ranged from r = 0.36to r = 0.8, indicating that while there may be some degree of variation, the relationship between grain length and TKW is still less impacted in different environments (Figure 3).

### 2.4. Influence of Climatic Variables on Grain Morphological Traits

**Figure 4:**
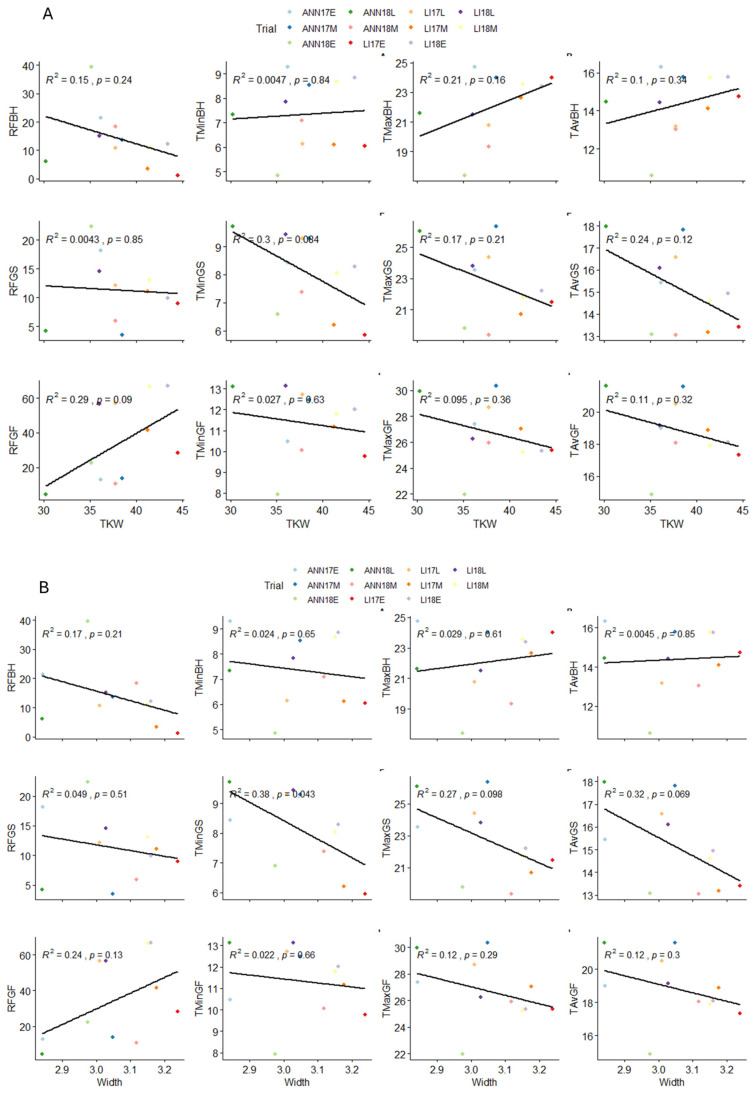

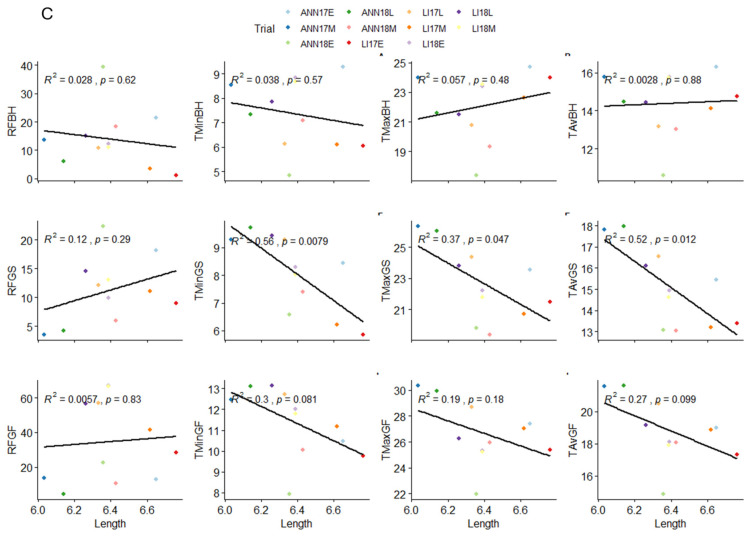
Linear regression analysis of environmental factors precipitation (RF), minimum temperature (Tmin), maximum temperature (Tmax), average temperature (Tav), and grain traits TKW (**A**), Width (**B**), and Length (**C**) across multiple growth periods, before heading (BH), grain setting (GS), grain filling (GF). The plotted values represent the average trait measurements across the eleven environments.

To explore the relationship between environmental variables and wheat grain development, we conducted a linear regression model using average, minimum, and maximum temperature and rainfall for the three main morphological parameters as compared to the three growth stages, (BH) 15 days before heading, (GS) grain setting 15 days after heading, and (GF) 30 days after heading across environments. Therefore, the average values across environments for both climatic variables and morphological parameters were used to identify which variable had the most significant impact on a particular trait. The results showed that minimum temperature during the grain setting stage had a significant negative influence on grain width, with a considerable impact of an R^2^ of 0.38, suggesting that lower minimum temperatures during this stage tend to result in wider grains, while for length, significant negative associations between temperature and grain length at multiple growth stages were detected. During the grain setting stage, minimum, maximum, and average temperatures exhibited significant negative impact, with values of R^2^ of 0.56, 0.37, and 0.52, respectively, indicating that higher temperatures during this stage tend to lead to shorter grain length. At same time, we found that both minimum and average temperatures during the grain filling period had significant negative impacts on grain length, with values of 71% and 62%, respectively. The relationship between environmental variables and TKW was not significant (Figure 4).

**Figure 5:**
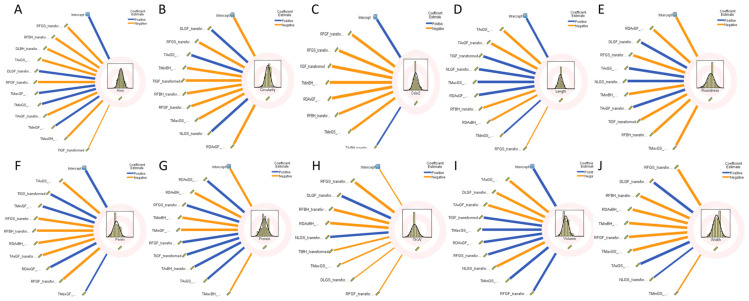
Factorial stepwise regression for significant climatic variables effects on each grain trait. (**A**) Area, (**B**) Circularity, (**C**) Color, (**D**) Length, (**E**) Roundness, (**F**) Perimeter, (**G**) Protein, (**H**) TKW, (**I**) Volume, (**J**) Width.

To further explore the impact of climatic variables on grain morphological traits and protein content, a factorial regression analysis was employed. This analysis incorporated eight environmental variables: rainfall (RF), average temperature (Tav), minimum temperature (Tmin), maximum temperature (Tmax), day length (DL), night length (NL), average radiation (RDav), and thermal inversion (TI). These variables were assessed at each growth stage of 249 winter wheat genotypes across 11 environments as shown in Figure 5. For grain area, the result revealed that rainfall during the grain setting stage had the most pronounced negative impact, accounting for a 25% variation, followed by the negative influence of rainfall before heading (15%) and day length (14%). Contrarily, the circularity of grain was positively affected by day length in the grain filling period contributing to 25% variation, followed by 18% for rainfall and 14% for average temperature in the grain setting stage. In terms of grain color, rainfall appeared as the primary influencing factor, accounting for 51% of the variation, with negative significant contributions during both the grain filling period (33%) and grain setting stage (18%); thermal inversion also negatively impacts the color with 15% in the grain filling period. Similarly, length was principally influenced negatively by the average temperature (51%) in the grain setting and grain filling period with 33% and 17%, respectively, followed by 13% for thermal inversion. Regarding the roundness, radiation and day length during the grain filling period (both 18%) influenced negatively, while rainfall during the grain setting stage (13%) had a positive effect. The analysis for grain perimeter showed that the average temperature in the grain setting period had a negative influence (15%), while thermal inversion during the same stage had a positive impact (15%), followed by minimum temperature in the grain filling period with 14%. Protein was most positively impacted by radiation in the grain setting period and negatively before heading with 19% and 13%, respectively, followed by rainfall in the grain setting period with 12%. In the case of TKW, rainfall during the grain setting stage had a negative impact (29%), while day length during the grain filling period had a positive influence of a similar percentage, followed by a negative impact of rainfall before heading with 16%. For the volume, the result showed that average temperature in the grain setting stage was the most influencing variable contributing to 27% of the negative variation, followed by negative impact (25%) of the day length in the grain filling period. Grain width was primarily influenced by rainfall in the grain setting stage with a significant negative effect (30%), followed by positive impact of the day length and negative for rainfall before heading with 15% each (Table S3).

### 2.5. GGE Biplots for Environment and Genotype Evaluation

**Figure 6:**
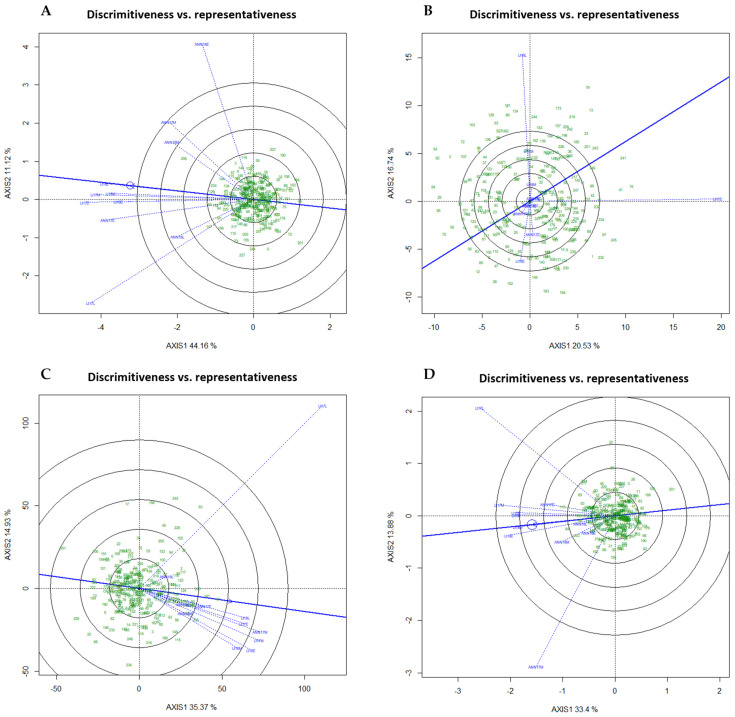
Discrimination and representativeness GGE biplots of the genotypic main effects of the WWAGI panel in eleven environments. Blue lines represent individual trials as location-year combinations. The average environment is indicated with a thick blue line with arrow passing through the biplot origin. Green numbers show individual entries. (**A**) Length, (**B**) Protein, (**C**) TKW, (**D**) Width.

To evaluate the environment and genotype, discriminativeness vs. representativeness and GGE biplots were conducted only for TKW, grain length, width, and protein content. LI17L and ANN18E showed the highest discriminating capacity for length, TKW, and grain width across the eleven environments. Regarding the protein, LI17E and LI18L were the most discriminating environments (Figure 6). The representativeness of every environment is indicated by the angle between the average environment axis and the environment line. Except for the protein content, all other traits were more representative, exhibiting a narrower angle. For length, ANN18E and LI17L were the least representative while it was LI17L for TKW and LI17L, ANN17M for width. Based on ‘which-won-where’ patterns in GGE biplots, environments were categorized into potential Mega-Environments (MEs), which will allow the identification of the best-performing and winning genotypes in each environment or group of environments 

**Figure 7:**
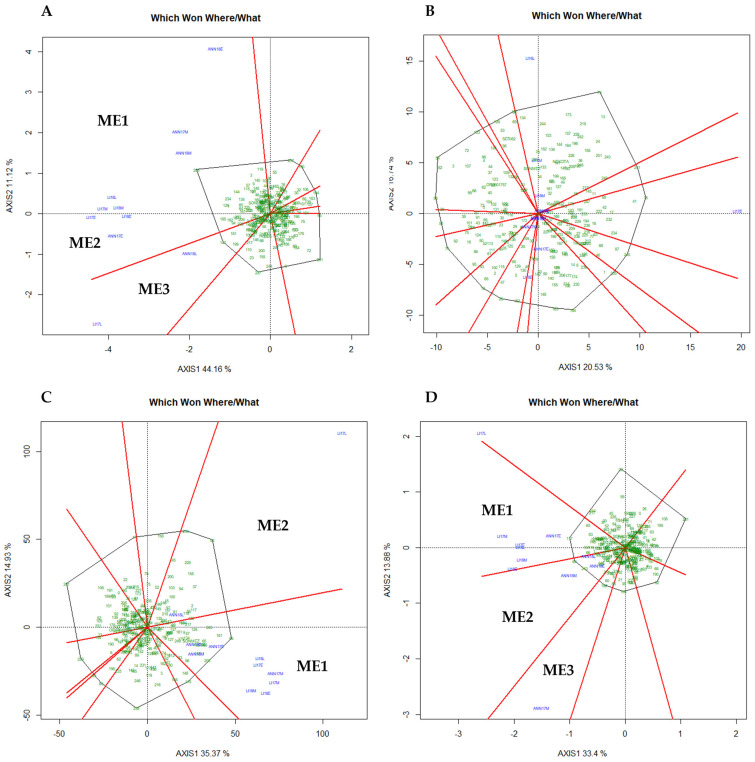
The GGE biplots show the “which-won-where” pattern of the WWAGI panel in eleven environments. Green numbers show individual entries. (**A**) Grain length, (**B**) Protein content, (**C**) TKW, (**D**) Grain Width.

**Figure 8:**
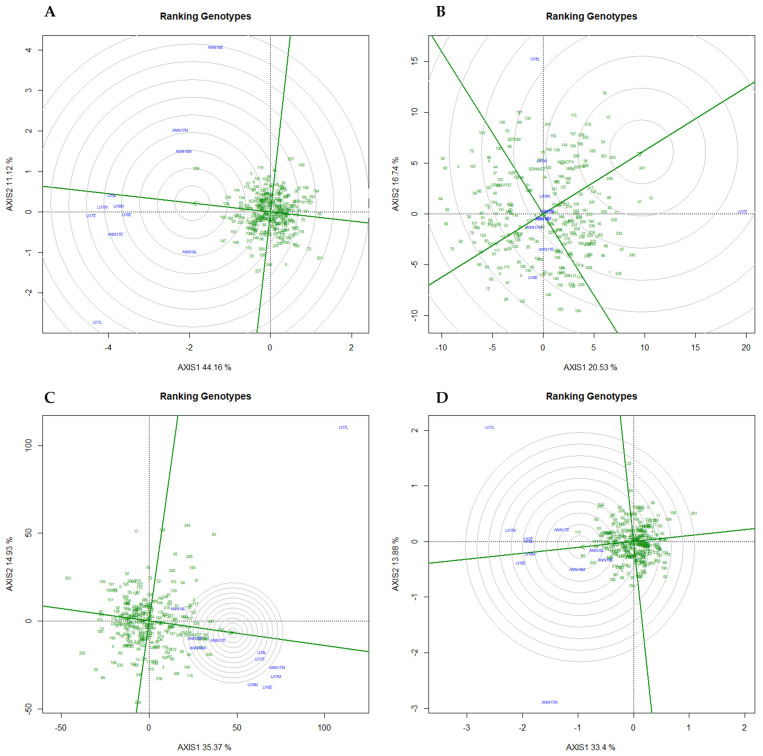
The GGE biplots showing the “Ranking genotypes” based on average performance and stability are displayed in a focused comparative biplot of the WWAGI panel in eleven environments. Green numbers show individual entries. (**A**) Length, (**B**) Protein, (**C**) TKW, (**D**) Width.

The GGE biplot captured 55%, 36%, 50%, and 47% of the total variation for length, protein, TKW, and width, respectively. Figure 7 shows genotype and GE interaction for every trial and environment clustered into groups with different winning genotypes. For length, the biplot suggests two MEs (Figure 7A); the first mega-environment (ME1) was represented principally by medium planting for both Annoceur (ANN) and Leida (LI) and during both years, the second mega-environment (ME2) was represented mainly by early planting, while the third mega-environment (ME3) was constituted from late planting. For TKW (Figure 7C), two MEs were identified, principally early and medium planting in the first mega-environment (ME1) and late planting in the second mega-environment (ME2). Regarding grain width (Figure 7D), the first mega-environment was represented primarily by Leida for the three sowing dates and two years. At the same time, the second mega-environment constituted of Annoceur during the cropping season 2018, while the third ME was represented by the medium planting of Annoceur in 2017. However, no clear pattern was detected for protein, suggesting that this trait was the most affected by the environment. According to Figure 8, genotypes 208, 147, and 185 were the best-performing genotypes for grain length in ME1, ME2, and ME3, respectively (Figure 8A). At the same time, for TKW, genotypes 84 and 181 were the best-performing in the ME2, while genotypes 208 and 64 were the best-performing for ME2 (Figure 8C). Regarding width, the best-performing genotypes were 117, 64, and 183 for ME1, ME2, and ME3, respectively (Figure 8D and Table S4).

### 2.6. Promising Genotypes in Diverse Mega-Environments

According to Hasan et al., Danakumara et al., Amelework et al., Wodebo et al., [23,24,25,26], and across different environments, an ideal genotype is demonstrating both high stability and a superior average performance. The ranking of a genotype’s performance is determined by its position close to the center of the concentric circles towards an absolute stable point (Average Environment Coordinate (AEC)). In our case (Figure 8), regarding TKW, genotypes 64, 181, and 243 were the most interesting as compared to the best of the four winter wheat cultivar checks (Nekota, OR9801757, Seri, and Sonmez). Genotype 64 showed a substantial 21% increase in the early planting, 18% in the medium planting, and an interesting increase with a value of 30% for the late planting, with a peak of 48% increase in LI18L, followed by 33% for LI17L. Similarly, genotype 181 demonstrated an increase of 11% for early planting, and 22% for both medium planting and late planting, with a maximum of 35% recorded in LI18L, followed by 34% in ANN17M. Genotype 243 showed an above-average increase of 14% for early planting, 15% for medium planting, and 22% for late planting, with a peak of 33% increase in ANN17M, followed by 32% in LI17L. 

For grain width, the most promising genotypes were 64, 117, and 243. Genotype 64 showed an increase of 7% for the early planting, 8%, and 10% for the medium and late planting, respectively. At the same time, genotype 117 exhibited a 4% increase in the early planting, and 10% for both medium and late planting, with the highest value of 17% registered in LI17L. Similarly, genotype 243 demonstrated an above-average increase of 6%, 8%, and 9% for early, medium, and late planting, respectively, with 17% as a maximum value recorded in ANN17M. Three outstanding genotypes were identified for grain length genotypes as the most promising: 208, 129, and 234. Genotype 208 demonstrated an increase of 9% for early planting, a remarkable increase of 17% for medium planting, and 8% for late planting as compared to the average of the four winter wheat cultivar checks (Nekota, OR9801757, Seri, and Sonmez), with 20% increase registered in LI18M followed by 19% for LI18L. Genotype 129 exhibited an above-average of 9% for the early planting, 8% for medium planting, and 7% for late planting as compared to the four checks, with a maximum of 13% increase registered in LI17L followed by 10% in LI18E. An increase of 7% for early planting, 14% for medium planting, and only 2% for late planting was observed for genotype 234 as compared to the four checks, with a maximum of 19% increase registered in ANN17M followed by 14% in ANN17E and ANN18E. For protein content, genotypes 241 and 243 emerged as the most promising. Genotype 241 showed an increase of 9% for early planting, 8% for medium planting, and 5% for late planting as compared to the average of the four winter wheat cultivar checks. At the same time, genotype 243 exhibited an improvement of 3%, 4%, and 5% for early, medium, and late planting, respectively.

### 2.7. Genome-Wide Association Study

GWAS for all traits studied identified 603 significant markers associated with traits of interest with LOD score of ≥3.0 across the eleven environments. These markers were distributed among the A, B, and D genomes, with 160, 243, and 200 markers, respectively. The significant markers were located on all chromosomes, with 2B carrying the highest number (70 MTAs) and 4D holding the lowest number (one MTA). The percentage of phenotypic variation explained by significant markers varied from 5% to 44%. The highest percentage was observed for grain color in LI18M, while the lowest value was detected for grain area. In total, 400 markers were associated with at least two traits each or with at least two environments. The highest number was detected within environment LI17M with 78 markers, while the lowest number was noticed within ANN17M with only one marker. Among these markers, AX-94824017 in chromosome 3D was the most significant association (LOD = 6.4) and responsible for 40% of the phenotypic variation for color. In addition, this marker was associated with two traits (color and circularity) in seven different environments (ANN17E, ANN18L, LI17E, LI17L, LI17M, LI18L, LI18M) with an average LOD score of 4.3 and 29% of the total phenotypic variation. Followed by marker AX-94910107 with an LOD score of 6.04, responsible for 39% of the phenotypic variation for color and present in five environments (LI17E, LI18L, LI17M, LI17L, ANN17E). At the same time, marker AX-94828467 present on chromosome 5D was associated with three traits (area, perimeter, and length) in four environments (LI17E, LI17M, LI18E, LI18L), with more than 15% of the phenotypic variation and an average LOD score of 3.7 (Table S4). Twelve markers were above the Bonferroni correction threshold (−log(*p*) = 5.2) associated with three traits (color, TKW, and roundness). Among these markers, nine were located on chromosome 3D, and present in five different environments. The highest number of markers were associated with color (10 MTAs), while TKW and roundness were associated with one marker each (Table 4).

**Figure 9:**
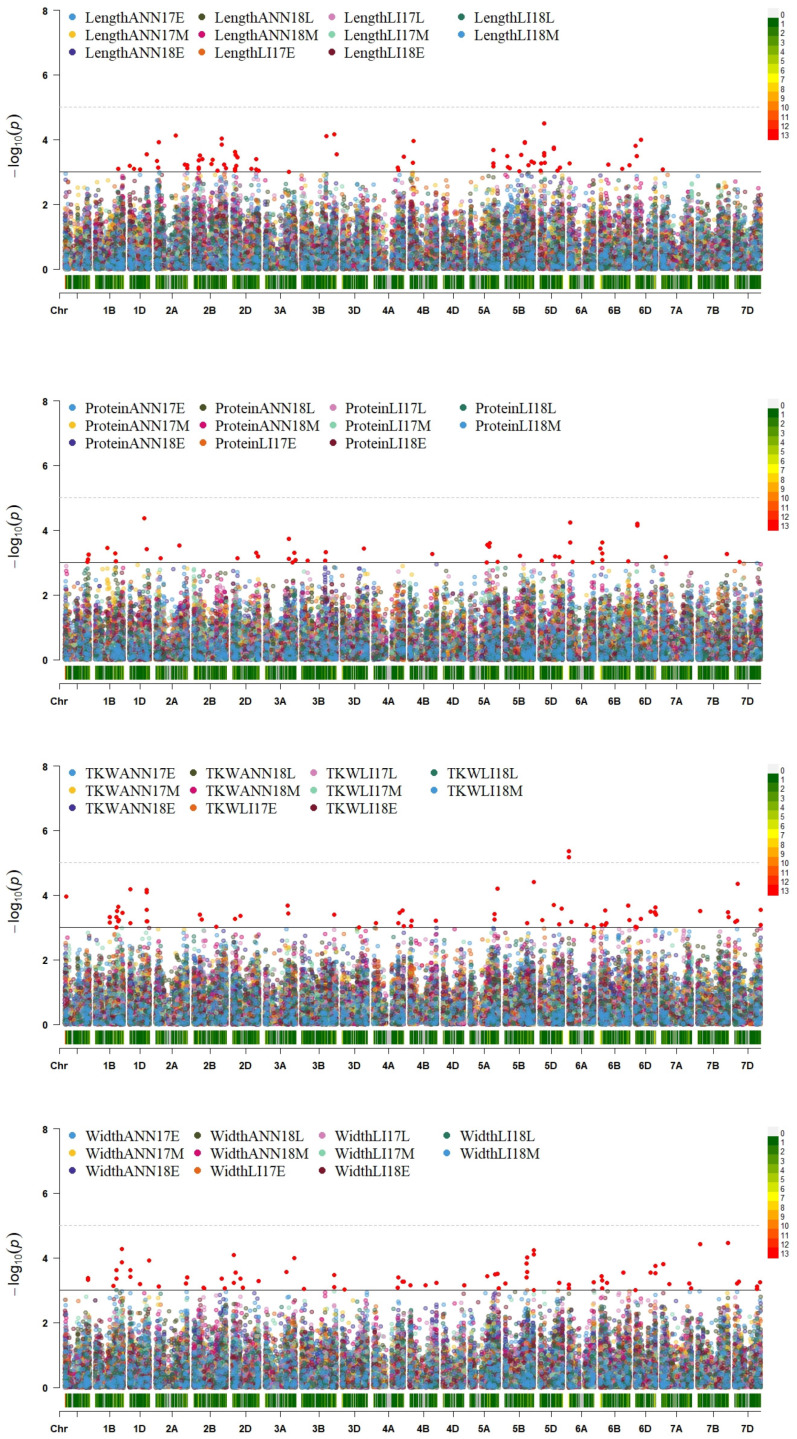
Genome-wide association scan for length, protein, TKW, and width of the WWAGI panel. Each environment is colored differently. The plots show Manhattan plots with significant SNPs. The X-axis is the genomic position of the SNPs in the genome alongside the SNP density, indicated by the color scale on the bar. The genome-wide scan −log10 (*p*-values) are shown on the Y-axis using common threshold (−log10(*p*) > 3) and Bonferroni correction (−log10(*p*) > 5.2).

Considering the four traits (grain length, width, TKW, and protein content), 275 significant MTAs were detected over all environments with 78 for length, 48 for protein, 72 for TKW and 77 for width. For grain length, the maximum number of significant markers (12 MTAs) was identified under environments ANN17M and ANN18E, while the lowest number was detected under ANN18L with only one marker. Similarly, for protein content, ANN17M contained the highest number of significant MTAs with nine markers, while LI18L presented the lowest number with only one marker. For TKW, fourteen significant markers were identified in LI17M as the maximum value followed by LI17L with twelve markers, while one marker was detected in ANN18L as the lowest number. Similarly, for grain width, the maximum value was detected within LI17M and LI17L with fourteen significant markers, while one marker was detected in ANN17M. Among these markers, 38 MTAs were associated with at least two traits each, with eleven markers associated in different environments (Figure 9 and Table S5). 

Interestingly, a total of 58 significant markers were simultaneously associated with grain length, width, and TKW across all environments with at least two of them at the same time. Out of these markers, ten were found to be collocated on chromosome 6D, comprising five markers linked to TKW, three to grain width, and two to grain length. Additionally, chromosome 1B revealed seven markers, of which four were detected for TKW, two for grain width, and one for grain length. Similarly, six markers were identified on chromosome 1D with three for TKW, two for grain width, and one for grain length, and five were detected on chromosome 5B with one for TKW for length and three for width. The remaining thirty significant markers revealed associations either with TKW and grain width or TKW and grain length (Table 5).

### 2.8. QTL–Environment Analysis 

To better understand the complex interactions between climatic variables and genetic markers governing key wheat traits, a comprehensive multi-environment correlation was conducted. The investigation examined the relationship between the allelic effect of significant markers and the climatic variables, which were assessed at fifteen-day intervals from sowing to harvest. The results revealed both newly identified and previously known genomic regions, with a specific focus on crucial grain traits including TKW, grain length, and grain width, which strongly influence yield. Twelve markers were identified to be correlated with climatic variables for TKW and either grain length or grain width (Table 6).

Co-locations on chromosome 6D were the most frequent with three markers for multi-trait loci controlling the mechanism of climatic variables response. Among these, marker AX-94459169 exhibited the highest absolute value of the marker effect reaction to a climatic variable for TKW (88%), exhibiting a negative impact of rainfall at heading date on TKW, indicating the critical role of precipitation timing in influencing grain weight development, while the lowest absolute value was noticed for marker AX-95107580 with 67%, indicating an adverse effect of thermal inversion on TKW (Table S6). Furthermore, for grain width, nine markers were present associated with both TKW and grain width, suggesting valuable information on the shared genetic regulation of these traits. Marker AX-95139913 exhibited the highest absolute allelic effect, demonstrating a negative impact (87%) of average maximum temperature at 60 days after sowing. This suggests that warmer temperatures during this specific period negatively influence the lateral dimensions of grains. At the same time, the lowest absolute value was observed for marker AX-95224161 (1B), revealing a negative impact on grain width of average maximum temperature at 135 days after sowing. Regarding grain length, the highest allelic effect was observed for marker AX-95127531 with 81%, indicating a negative impact associated with the average temperature during the heading date.

**Figure 10:**
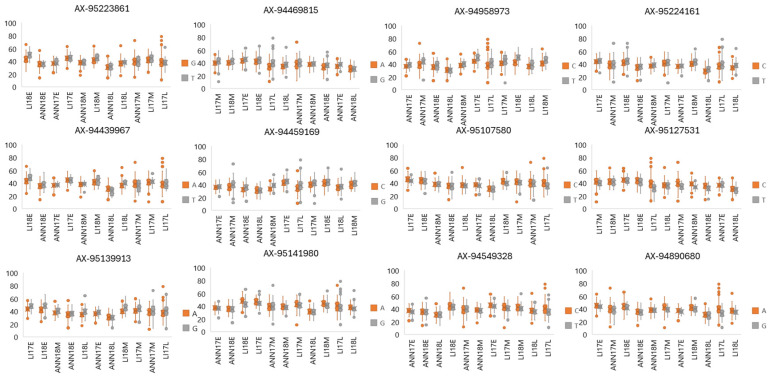
Genetic dissection of the allelic effect at the identified significant markers correlated with climatic variables for TKW, grain width, and length across all environments. The X-axis indicates the environments under this study. The Y-axis represents the TKW values for each allele and environment.

The results of the genetic dissection for the allelic effect at selected significant markers significantly correlated with climatic variables across environments for TKW, grain width, and length are presented in Figure 10. The genetic influence of allele T at marker AX-95223861 revealed a positive effect on TKW and grain width under high temperatures. At the same time, the effect of allele G at marker AX-94459169 exhibits a positive impact on TKW and grain width under drought conditions, while allele G at marker AX-94469815 showed a positive impact on TKW and grain width under long days and warmer temperatures. At marker AX-94958973, allele T presented a positive effect on TKW under low temperatures during the heading period. Allele C at marker AX-95127531 showed a negative impact on grain length under high temperature. 

## 3. Discussion

Wheat is a staple crop of global importance for a large portion of the worldwide population, contributing to up to 20% of its daily intake. In recent years, wheat breeders and scientists have achieved slow yield progress, and the yield plateau has become an alarming threat to food security. Wheat quality is a complex concept, including grain morphology and nutritional composition. Grain morphological traits are some of the most important quality characteristics due to their significant impact on grain weight, flour yield, and market value [27]. However, the negative relationship between protein content and grain yield components in wheats has limited the development of both traits [28,29]. The notable heritability of a wide range of grain quality traits in wheat renders it highly amenable to DNA marker selection application. This technique allows for the simultaneous selection of varieties with high yield, as assessed through conventional field testing, alongside those with superior grain quality. Furthermore, resilience is vital for adapting to variable weather conditions. Aligning with this goal, our study is dedicated to the effective identification of markers that denote resilience in wheat, specifically relating to grain morphology and protein content. In the current study, we used GEI to understand grain morphology and protein content changes and performance of the genotypes (advanced lines and varieties) in winter wheat multi-environment trials. In our population, genotypes showed a significant level of variation for morphological grain size, TKW, and protein content with moderate to high heritability. In agreement with many previous studies, there was a significant strong positive correlation between grain morphological traits, and negative when they were compared to protein content [30,31]. Elsewhere [32], a significant negative correlation between TKW and protein content in a population of 194 of bread wheat was observed. Moreover, a strong positive correlation between grain size and weight in a population of 231 synthetic wheats has been reported [27]. Gao et al. [33] reported a strong correlation between grain area, perimeter, length, width, and TKW. Simmonds et al. [34] revealed that a mutation in *TaGW2-A1* resulted in an increase in grain width and length, which in turn increased thousand kernel weight. The relationship between these traits, linking positively grain morphological characteristics and grain weight, and negatively with protein content, suggest that longer and wider grains have the capacity to store more starch and carbohydrates, which increases grain weight and results in the dilution effect for protein content. Kenzhebayeva et al. [35] reported a low association between grain shape and protein content using wheat transformed by radiation. The highest mean of grain length, width, and TKW was recorded in LI17E, the environment with the lowest rainfall and the highest number of days, as compared to ANN17 and ANN18, but with good monthly distribution, suggesting that this environment could be considered as favorable cropping season for these genotypes in relation to rainfall. At the same time, the late planting in ANN18 and LI17 exhibited the highest value for protein content, while all other traits showed a significant decrease from the early planting to the late planting. This increase in protein can be attributed to the dilution effect under the low-stressed conditions in the early and medium planting dates due to an increase in carbohydrates in the grain as suggested by the higher TKW. According to the ANOVA table, genotype, environment, and GE interaction were highly significant in all trials. An exception was noted for grain color, which was not influenced by GE interaction. This suggests that the ranking and the selection for grain color remain stable across locations and environments. Similarly, Zhao et al. [36] revealed no effect of genotype by environment on grain color using seventy-nine diverse spring wheat genotypes where the environment had the lowest impact on genotypic performance for color with only 17% of the total variation. The GEI significantly contributed to the variation in protein content, accounting for 60% and representing a tenfold greater effect than the genotype. Similarly, for length, width, and TKW, the GEI also captured the highest value of variation with 37%, 44%, and 45%, respectively, with moderate influence as compared to the environment and genotype effect. This pattern suggests that the genotype by environment interaction accounts for the highest source of variation for protein content and therefore drives protein content variability for our population. At the same time, the GEI followed by environment represents the main component of variation for width and TKW. The genetic influence is almost as significant as the environment and GEI when it comes to length. Similar research results about the comparative importance of environment, genotype, and GEI were reported [6,37,38,39]. The substantial interaction observed between the genotype and environment results in reduced grain morphological traits and protein content stability in our panel. Thus, it is advised for wheat breeders to increase the number of genotypes in a mega-environment to identify the stable genotypes with better grain shape and optimal quality.

The Mediterranean area, which includes Spain and Morocco, is renowned for having a very variable climate within and between the seasons. Therefore, more advanced statistical techniques than the conventional ANOVA are needed for a thorough assessment of GE interactions. Based on the GGE biplot analysis, a Mega-Environment (ME) can be determined when different genotypes are adapted to distinct groups of environments and the variance between groups is higher than within groups [23,24,25,37,40]. In our study, the different environments tested were clustered into two MEs for TKW and three for grain length and width. This result is in agreement with the result reported by [41] where two winter wheat MEs in Ontario were suggested after examining the ME analysis and test site for winter wheat in Canada. Similar conclusions were made by [42] in relation to winter wheat tested at eight locations who found between three and four MEs for grain yield. However, for protein content, the targeted region was not better presented as MEs but rather as a single environment, indicating that the evaluation of genotypes for this specific trait must be performed over years and locations. Therefore, the selection of future varieties adapted to the Moroccan and Spanish environments must prioritize genotypes with wide adaptability rather than specific adaptations or high protein concentration. The results of this study indicate that there was one or more promising genotype(s) for each group of MEs as the winning genotype(s) except for protein which was different every year. Genotypes 208, 129, and 234 were consistently the winning genotypes for grain length, while 64, 117, and 243 were the promising genotypes for grain width and genotypes 64, 181, and 243 for TKW. The definition of a ME states that a sufficient number of locations is necessary to confirm that genotype responses are consistent across locations within a ME [37]. However, in the case of protein, this study’s limitation to just two locations, each with three sowing dates, made it difficult to draw definitive conclusions. Consequently, for more conclusive evidence of these MEs, future studies are recommended to include a broader range of locations. A study [32] reported an unstructured GEI for grain protein content in 194 F7 RILs of bread wheat. Altogether, these results suggested that the behavior of these traits can be complex and unpredictable across different environmental conditions and sowing dates. This further supports the need for extensive research across varied locations to decode the complexities of genotype and environment interactions.

Climatic variables and weather conditions including temperature and rainfall during the heading date had probably the most significant influence on grain yield and quality. It is also responsible for controlling the adaptation of wheat to a broad range of environments [43,44,45]. Many key climatic variables influencing wheat growth development under Mediterranean climates in Morocco and Spain varied between the eleven environments used in this study. It has been shown that minimum and maximum temperatures during growth stages, rainfall, terminal heat stresses, and late frost are key climatic factors affecting wheat adaptation in Mediterranean environments [46]. For this scope, the impact of the climatic variables was assessed between and within each environment. Across environments, a high minimum temperature during the grain setting stage was identified as the key climatic variable negatively impacting grain width, while, for grain length, a significant negative relationship was detected with minimum, maximum, and average temperatures during the grain setting and filling stages. Moreover, our findings highlighted a greater influence of GEI on grain width compared to grain length. This could be attributed to the association of grain width with the plant’s ability to effectively fill the grain, indicating a higher sensitivity of grain width to environmental variations, which may play a crucial role in shaping the lateral dimensions of developing grains. While grain length exhibits a relatively less sensitive response to environmental conditions, reflecting a more constitutive and stable trait, the relationship between climatic variables and TKW was not significant. However, using the factorial regression analysis, a negative effect of the average temperature on grain perimeter before heading raises the possibility of a relationship between lower temperatures and constrained grain spatial expansion. This indicated that heading is preceded by a temperature-sensitive period, during which the grain’s structural development is influenced by lower temperatures, potentially changing the grain’s overall size and shape. Furthermore, rainfall appeared as a key factor, negatively influencing grain area, color, width, and TKW during the grain setting stage. This may result in decreased grain expansion due to different sowing dates, which restricts roots from receiving enough key nutrients necessary for proper grain setting interfering with vital metabolic processes that facilitate grain expansion. Several authors suggested that the stem-elongation stage to anthesis and post-anthesis including the grain setting stage are the most sensitive times [47,48,49]. However, ref. [50] reported that drought after flowering resulted in a 5.2% decrease in TKW and 20% reduction in grain number using a bread wheat cultivar, while for the grain filling period, a dominant role of rainfall and average temperature negatively impacted grain color and grain length, respectively, indicating that high average temperature resulted in accelerating some metabolic processes, which might impact grain elongation. Conversely, a positive influence of day length on grain circularity, TKW, and width highlights the critical role of day length in producing rounded and heavier grain. The grain filling period has been subject to many studies to determine the environmental factors influencing grain shape and weight in wheat [16,17,51,52]. This is in agreement with the result reported by [44,53,54] where they showed that different genotypes tended to stop grain development early and accelerate physiological maturity when temperatures increased during the grain filling period resulting in diminished final grain weight.

Given that GEI often impacts genetic analysis used to identify the genomic area for quantitative parameters across many environments, it is an essential criterion to assess the significance and dependability of certain loci. In our study, GWASs for grain shape, TKW, and protein content were conducted for the WWAGI population in each environment independently. Overall, 603 significant MTAs with LOD score of ≥3.0 across eleven environments were identified on all chromosomes. However, the most important MTAs are those identified for grain length, width, TKW, and protein content (275 MTAs) due to their immediate effect on improving grain yield and quality. In the current analysis, seventy-eight MTAs were associated with at least two traits each, with eleven markers associated in different environments. Numerous studies have reported mapping for grain weight and shape in wheat, but there are few that explain the variation and stability of grain weight, size, and shape across several environments [27,55,56]. The most important QTLs for grain length, TKW, and width were mapped on chromosomes 6D, 5D, and 2D [56]. However, in this study, significant SNPs on all other chromosomes, except 1A, 3D, 5A, 6A, 7B, and 7D for length, 1A, 2A, 3A, 3D, 4B, 5D, and 7D for TKW, and 3D, 4B, 5D for width, were identified. Chromosomes 2B and 5D exhibited the highest number of significant SNPs, each with seven SNPs for grain length. Regarding TKW, the highest number was identified on chromosome 6D and 1D with six SNPs each, while for grain width five significant SNPs were detected on chromosome 5B. Interestingly, we found nine chromosomes (1B, 1D, 2B, 2D, 3B, 4A, 5B, 6B, 6D) that showed an overlapping of significant SNPs with the three traits at the same time indicating these regions as stable. This is in agreement with the result reported [27] indicating a co-linearity of MTAs for different grain morphology traits on chromosomes 1A, 2B, 3A, 3D, and 5B with a complete region on chromosome 2B (51–69.9 cM) exhibiting 31 significant MTAs. Similarly, [57] reported a QTL on chromosome 1D for the grain length, width, and weight, while none of the other QTLs detected for grain length and width overlapped with grain weight. Furthermore, previous research on wheat RIL populations by [58] indicated an association between chromosomes 4D and 7D and grain width. In addition, QTLs for grain weight on chromosomes 1D, 2D, 5D, and 7D were reported by [32,55]. In our study, 130 significant MTAs affecting grain size and shape for more than two traits and/or environment have been identified across all chromosomes, and many of them were found within the same regions, suggesting that they may have novel allelic variability for grain size and shape and underlying the importance for meta-QTL identification for grain size, shape, and weight. Several QTLs and meta-QTLs were previously reported for grain weight, width, and length on different chromosomes. Zhang et al. [59] reported a meta-QTL for grain weight on chromosome 2D. Campbell et al. [60] highlighted QTLs on chromosomes 1A, 2A, 2B, 2D, and 3D, and chromosome 6D; [61] revealed a group of QTLs for grain length, width, and weight. Wheat grain color has been subject to many studies identifying three genes located on the long arm of homoeologous 3 (3AL, 3BL, and 3DL chromosomes) controlled by the R-1 (red) genes as the genes governing wheat grain color [62]. Similarly, our study identified 37 significant MTAs on chromosomes 3A and 3D, with the majority (34 MTAs) identified on 3D. 

The genetic dissection conducted in this study highlights the relationship between specific (SNPs) and climatic variables, illuminating their impacts on key morphological grain traits. The highlighted relationships play a crucial role in different developmental stages, ranging from the early vegetative to the late grain-filling period. Notably, the increase in grain width due to the negative effect of allele T at marker AX-95224161 when the average maximum temperature is low highlights the sensitivity of this developmental stage to high temperatures. This result is consistent with previous research that shows how temperature negatively affects grain size during grain filling [63,64]. When subjecting spring wheat to both day and night warming by changes in sowing date and additional infrared heating, [65] observed a 3% reduction in grain weight for every degree Celsius of post-anthesis mean temperature increase. Furthermore, allele A at marker AX-95141980 demonstrates a strong negative effect on TKW under long days, suggesting that photoperiod sensitivity plays a major role in determining grain weight and highlighting the significance of longer day lengths in affecting the length of the reproductive phase. Some studies [66,67] highlighted the relationship between shorter daylength and the increased number of spikelets per spike independently of the temperature, which may result in increased grain number. However, a negative association has been generally reported between TKW and grain number in wheat [68,69]. The genetic effect of allele G at marker AX-94459169 exhibits a positive impact on TKW and grain width under drought conditions. This marker could then be used, after validation, to increase grain width and therefore TKW and potentially flour yield under drought. A high negative correlation between yield and heading date under irrigated and rainfed environments was reported by [70].

This study dissects the intricate relationship between genetic factors and environmental conditions in wheat modern and advanced lines, particularly focusing on grain quality traits such as grain morphology and protein content. With the significant heritability of these traits, the application of DNA markers emerges as a promising strategy, enabling the simultaneous selection of varieties that achieve both high yield and superior grain quality. The research underscores the importance of resilience, supporting the development of wheat varieties capable of adapting to variable weather conditions given the challenge of enhancing both protein content and yield simultaneously. The substantial role of genotype–environment interaction in trait variability highlights the dynamic relationship between genotypes and their environmental contexts, further emphasizing the need for a deep understanding of Mega Environments in selecting broadly adaptable wheat varieties. Insights from genome-wide association studies (GWASs) offer valuable markers associated with key traits, providing targets for breeding programs. Additionally, the study’s exploration of how climatic variables like temperature and rainfall affect grain traits offers novel perspectives for breeding wheat varieties tailored to specific environmental challenges. Altogether, these findings mark a significant step forward to optimizing wheat quality and yield, ensuring the crop’s resilience and adaptability across diverse growing conditions.

## 4. Materials and Methods

### 4.1. Plant Material

**Figure 11:**
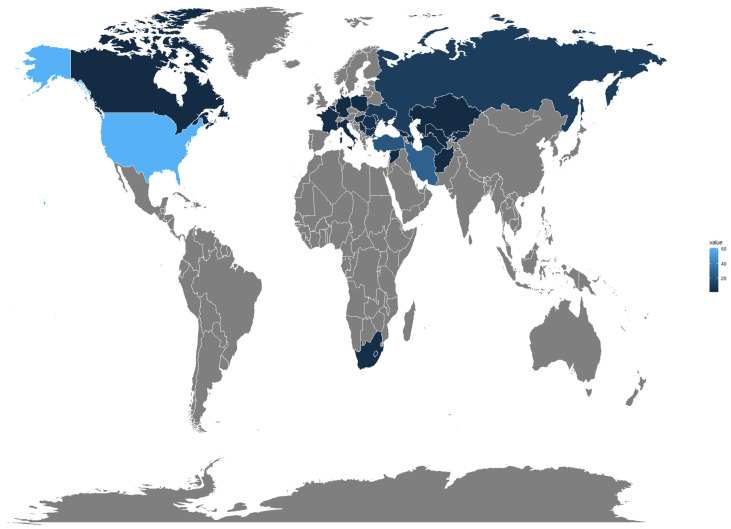
Geographic origin of the Winter Wheat Association Genetics Initiative (WWAGI) panel with 249 advanced lines and varieties used.

A panel consisting of 249 advanced lines and varieties from the Winter Wheat Association Genetics Initiative (WWAGI) developed by the International Maize and Wheat Improvement Center (CIMMYT) was used in this study. The panel was previously described by El Baouchi et al. [71]. The lines originated from thirty-two different countries and represent six distinct regions, Central and Western Asia, Europe, North America, CGIAR. As a result, diverse agro-ecological zones are represented. The highest number of genotypes was sourced from CGIAR (CGR) with 65 genotypes, followed by the United States of America (USA) with 61 and Iran, Turkey, Russia, Ukraine, Bulgaria, and Moldova with 27, 22, 10, 9, 6, 5, respectively (Figure 11).

### 4.2. Experimental Design and Area of Study

The WWAGI genotypes were grown during the cropping seasons 2016–2017 and 2017–2018 at the at Annoceur (ANN) INRA research station (33°41′05.2″ N 4°51′19.9″ W) in the highlands of Morocco and at Lleida (LI) in the north-east of Spain (41°40′ N 00°20′ E). Experiments were planted in an augmented design using four checks (NEKOTA, SERI82, SONMEZ, and OR9801757) at three planting dates, Early (EP), Medium (MP), and Late (LP) in November, December, and January, respectively. Pests, diseases, weeds, and fertilization were managed according to the best usual agronomic practice.

### 4.3. Environment Characterization

Daily data recorded from weather stations located at each site were used to calculate averages of environmental variables for each planting date (Table 6). The following variables were measured from sowing to harvesting, daily average, minimum, and maximum temperatures (Tmin, Tmax, °C), reference evapotranspiration (ET0, mm), and average maximum and minimum daily relative air humidity (Rhmax, Rhmin, %). In addition, the difference of day length (DiffDaylength) was calculated for every 15 days by subtracting the minimum value of day length from the maximum value of day length. Similarly, the accumulated day length (AccumDaylength) was computed by accumulating the difference of day length. Furthermore, a climate matrix was developed for every environment with daily average, minimum, and maximum and values were divided into three growth stages, (BH) 15 days before heading, (GS) grain setting 15 days after heading, and (GF) 30 days after heading [72,73].

### 4.4. Grain Morphology and Protein Content

The morphological parameters and protein content characterization were performed at ICARDA Cereal and Legume Quality Laboratory. The Grain samples obtained from twelve spikes of every plot were all scanned using a flatbed scanner (CanoScan LiDE 220, Canon U.S.A., Inc. One Canon ParkMelville, NY 11747, USA). The images were processed for analysis using Grainscan software developed by CSIRO (https://data.csiro.au/collection/csiro:8827 accessed on 2 February 2024) [74] and data were generated for every grain for area (mm^2^), perimeter (mm), length (mm), width (mm), and color (Color2) which represents the green and magenta of the color a*. Additionally, the Thousand Kernel Weight (TKW g) was calculated based on the number of grains and the weight of the scanned dry grain and the roundness was computed by dividing the width by the length. Furthermore, the protein content for every plot was assessed by the Near Infrared spectroscopy (NIRS, Foss DS2500, Allé 1, DK-3400 Hilleroed, Denmark) method using an in-house calibration developed specifically for bread wheat.

### 4.5. Genotyping

The 35K Axiom^®^ Wheat Breeder’s Array (Affymetrix UK Ltd., Cheshire, UK) was used to genotype the WWAGI panel as per manufacturer’s guidelines [75] with a total array of 35,143 SNP markers. The protocol, genetic diversity, and population structure were described previously by [71]. After the elimination of markers with minor allele frequencies (MAFs) less than 5% and markers with more than 10% missing data, 11,476 Axiom SNP markers were left in the refined set.

### 4.6. Statistical Analysis

Statistical analysis was performed using the RStudio spatial (row × col) analysis StatgenSTA package for each environment. The resulting table of Best Linear Unbiased Estimates (BLUEs) was used to model the GEI using “StatgenGxE” package (https://github.com/Biometris/statgenGxE/, accessed on 29 February 2024) to test the significance effect of genotype and environment. The R studio “GGEBiplotGUI” package was used for GGE biplots. The graphical GGE biplot was used to explain G × E interaction and genotype, environment ranking based on mean and stability. The GGE biplot graphs are based on genotype evaluation (mean vs. stability), multilocation assessment (which won where), and winning genotype ranked for each trait per mega-environment. Singular-value partitioning = 2, transformed (transform = 0), environment-centered (centering = 2), and standard deviation standardized (scaling = 0) were used to create the biplots. Person correlation coefficients between all traits were computed and tested for their statistical significance using prcomp function. The PCA and heatmap were generated using RStudio visualization of corrplot and ggplot packages, integrated into the R software. Furthermore, SPSS V20 was used to evaluate the effect of climatic parameters on each trait during the three stages (BH, GS, and GF).

### 4.7. Genome-Wide Association Study (GWAS)

The genetic diversity and structure described by [71] was used to genome wide association (GWAS) analysis. The filtered SNPs, gene-specific marker results, and the BLUEs for each trait in individual environment were used for the GWAS using GAPIT V3 (Genomic Association and Prediction Integrated Tool) in R [76]. Three models were performed; GLM [77], MLM [78], and CMLM [79] (https://github.com/jiabowang/GAPIT3 (accessed on 23 February 2024)). In addition to PCA, the relative kinship analysis was carried out to identify the genetic relationship between genotypes using the VanRaden method implemented in GAPIT [80,81]. Marker trait associations (MTAs) were declared if their −log10(*p*) value was higher than 3.0. The significance threshold for MTAs was elaborated based on Bonferroni correction of *p* ≤ 0.05/n, where n is the number of SNP markers used. A Q-Q plot with predicted vs. observed log10(*p*) value was used to determine the quality of the fitted GWAS model. Manhattan plots was drawn by the CMplot R package (https://cran.r-project.org/web/packages/CMplot/index.html (accessed on the 7 February 2024)) to visualize the MTAs. The QTL–Environment analysis was performed using the phenotypic data of the traits collected from individual environments, across multiple environments and their Best Linear Unbiased Estimates (BLUEs), to identify traits associated with QTLs.

## 5. Conclusions

Wheat is a vital crop ensuring food security worldwide. The present work was conducted to characterize, evaluate, and explore the WWAGI panel across eleven environments for morphological traits and protein content, identifying genotypes with high performance and stability, and evaluate the impact of climatic variables on the different traits. Wide ranges of genotypic variability were present in the panel for all measured grain traits. The strong correlation between morphological traits suggested the possibility of improving simultaneously. Significant GEI interaction was found. Genome-wide association in the WWAGI showed significant associations for grain traits. Overall, 603 significant MTAs with LOD score of ≥3.0 across eleven environments were identified on all chromosomes. To validate the relative significance of the chromosomal regions linked to grain size, more research must be carried out. The results obtained would be an important asset for developing and implementing targeted breeding programs aimed at improving grain size, weight, and quality, alongside effective performance and adaptation in different sowing dates across the Mediterranean areas. 

## Figures and Tables

**Table 1:** Localization and description of the environments included in this study.

Site	ANN17	ANN18	LI17	LI18
Trial identification	33°41′05.2″ N, 4°51′19.9″ W		41°40′ N, 00°20′ E	
Environmental conditions for Early planting (EP)				
Sum ET0 (mm)	633.7	-	580.08	694.71
Average Tmin (°C)	6.88	2.94	5.82	4.64
Average Tmax (°C)	21.9	16.47	17.99	17.74
Average Rhmin (%)	48.2	59.58	-	72.62
Average Rhmax (%)	55.98	66.6	-	94.12
Sum Rainfall (mm)	1060.7	713.4	295.3	263.5
Average Daylength (H)	12.03	11.72	11.72	11.69
Average Nightlength (H)	11.96	12.27	12.28	12.03
Environmental conditions for Medium planting (MP)				
Sum ET0 (mm)	600.1	-	565.3	664.8
Average Tmin (°C)	5.06	3.05	6.51	7.37
Average Tmax (°C)	21.18	16.52	19.3	20.27
Average Rhmin (%)	52.79	68.1	-	72
Average Rhmax (%)	61.08	75.05	-	93.96
Sum Rainfall (mm)	184.8	628	231	260.9
Average Daylength (H)	12.08	12.2	12.05	12.12
Average Nightlength (H)	11.92	11.8	11.95	11.88
Environmental conditions for Late planting (LP)				
Sum ET0 (mm)	-	-	553.05	643.88
Average Tmin (°C)	-	5.25	7.48	8.26
Average Tmax (°C)	-	18.32	21.5	21.84
Average Rhmin (%)	-	69.36	-	69.96
Average Rhmax (%)	-	76.18	-	93.68
Sum Rainfall (mm)	-	494.2	223.4	237
Average Daylength (H)	-	12.89	12.63	12.98
Average Nightlength (H)	-	11.1	11.37	11.02

**Table 2:** Descriptive statistics of morphological traits and protein content of the Best Linear Unbiased Estimates (BLUEs) of the Winter Wheat Association Genetics Initiative (WWAGI) panel used at each environment.

Trial	ANN17		ANN18		LI17		LI18	
Variable	Mean	SD	Mean	SD	Mean	SD	Mean	SD
Early planting (E)								
Area	14.83	1.22	15.05	1.74	17.28	1.44	15.89	1.5
Circularity	0.45	0.02	0.5	0.04	0.51	0.02	0.51	0.02
Color	126.51	9.95	133.85	9.59	136.89	9.67	127.32	8.04
Length	6.65	0.35	6.36	0.38	6.76	0.31	6.39	0.31
Perimeter	20.28	0.95	19.34	1.03	20.69	0.91	19.88	0.93
Protein	12.19	0.81	12.82	0.39	12.19	1.25	12.29	0.97
Roundness	0.43	0.03	0.47	0.03	0.48	0.02	0.5	0.03
TKW	36.19	4.65	35.14	6.8	44.55	5.58	43.45	6.9
Volume	6.06	0.56	5.3	0.51	5.31	0.44	4.93	0.45
Width	2.84	0.15	2.98	0.19	3.24	0.16	3.16	0.19
Medium planting (M)								
Area	14.58	1.82	15.98	1.48	16.59	1.64	15.89	1.46
Circularity	0.52	0.02	0.51	0.02	0.5	0.02	0.51	0.02
Color	126.55	9.37	132.64	9.63	131.81	8.39	129.28	7.01
Length	6.03	0.35	6.43	0.35	6.62	0.32	6.39	0.31
Perimeter	18.93	1.03	19.72	0.98	20.32	0.95	19.72	0.94
Protein	12.94	0.99	13.24	0.21	11.57	1.06	12.52	1.08
Roundness	0.51	0.04	0.49	0.03	0.48	0.03	0.5	0.03
TKW	38.52	9.12	37.75	5.27	41.23	7.27	41.47	6.57
Volume	4.59	0.5	5.05	0.45	5.24	0.44	4.93	0.42
Width	3.05	0.26	3.12	0.16	3.18	0.2	3.15	0.18
Late planting (L)								
Area	-	-	13.77	1.54	15.06	2.08	14.95	1.45
Circularity	-	-	0.49	0.02	0.5	0.02	0.51	0.02
Color	-	-	128.62	9.72	121.88	11	125.82	8.15
Length	-	-	6.14	0.31	6.33	0.4	6.26	0.32
Perimeter	-	-	18.63	0.92	19.4	1.22	19.28	0.92
Protein	-	-	14.28	0.41	14.25	0.44	12.81	1.13
Roundness	-	-	0.46	0.03	0.48	0.03	0.49	0.02
TKW	-	-	30.23	6.09	37.61	9.67	36.02	6.11
Volume	-	-	5.23	0.44	5.16	0.43	4.98	0.41
Width	-	-	2.84	0.2	3.01	0.25	3.03	0.18

**Table 3:** Analysis of variance for morphological trait and protein content of the WWAGI across 11 environments.

Trait	Fixed Term	d.f	Sum of Squares	Chi-Sq Prob	%TSS
Area	Environment (E)	10	2259.76	<0.001	29%
	Genotype (G)	252	2138.46	<0.001	28%
	GE interaction	2299	3303.75	<0.001	43%
Circularity	Environment (E)	10	7288.65	<0.001	53%
	Genotype (G)	252	2318.66	<0.001	17%
	GE interaction	2299	4089.96	<0.001	30%
Color2	Environment (E)	10	870	<0.001	17%
	Genotype (G)	252	2082.64	<0.001	40%
	GE interaction	2299	2243.19	0.794	43%
Length	Environment (E)	10	3325.37	<0.001	30%
	Genotype (G)	252	3599.8	<0.001	33%
	GE interaction	2299	3990.61	<0.001	37%
Perim	Environment (E)	10	2728.26	<0.001	29%
	Genotype (G)	252	3115.05	<0.001	33%
	GE interaction	2299	3565.56	<0.001	38%
Protein	Environment (E)	10	2711.6	<0.001	34%
	Genotype (G)	252	508.02	<0.001	6%
	GE interaction	2297	4841.68	<0.001	60%
Roundness	Environment (E)	10	6210.9	<0.001	44%
	Genotype (G)	252	3219.73	<0.001	23%
	GE interaction	2299	4701.13	<0.001	33%
TKW	Environment (E)	10	1597.97	<0.001	27%
	Genotype (G)	252	1644.63	<0.001	28%
	GE interaction	2287	2679.47	<0.001	45%
Volume	Environment (E)	10	8402.18	<0.001	43%
	Genotype (G)	252	5409.19	<0.001	28%
	GE interaction	2299	5790.41	<0.001	30%
Width	Environment (E)	10	2470.96	<0.001	34%
	Genotype (G)	252	1597.06	<0.001	22%
	GE interaction	2299	3182.64	<0.001	44%

d.f: degree of freedom; %TSS: percentage relative to total sum of squares.

**Table 4:** Significant marker associations that pass the Bonferroni correction threshold.

Environment	Trait	Marker	Chromosome	Position	−log10(*p*)	R^2^
LI17E	Color	AX-94824017	3D	572188094	6.399121	0.40
LI17E	Color	AX-94910107	3D	572478517	6.058349	0.39
LI17E	Color	AX-94748732	3D	572154318	5.949095	0.39
LI18L	Color	AX-94824017	3D	572188094	5.727154	0.38
LI17E	Color	AX-94415259	3D	572708647	5.716138	0.39
LI17E	Color	AX-95120609	3A	705206286	5.470853	0.38
LI17E	Color	AX-94721213	3D	572837198	5.386912	0.38
ANN18M	TKW	AX-94394209	6A	1717618	5.357748	0.13
LI18L	Color	AX-94721213	3D	572837198	5.355953	0.37
LI18E	Roundness	AX-95156025	2B	148607565	5.322127	0.19
LI17E	Color	AX-95124645	3D	572704056	5.233392	0.38
LI17M	Color	AX-94824017	3D	572188094	5.20292	0.43

**Table 5:** Significant markers associated to TKW, grain width, and grain length.

Environment	Trait	Marker	Chromosome	Position	−log10(*p*)
LI18E	Length	AX-94407122	1B	564644617	3.09
LI17M	TKW	AX-94407122	1B	564644617	3.63
LI17E	TKW	AX-94804372	1B	353759591	3.16
ANN18M	TKW	AX-94804372	1B	353759591	3.32
LI18L	TKW	AX-95224161	1B	518659990	3.32
LI18M	Width	AX-95224161	1B	518659990	3.36
LI18L	Width	AX-95224161	1B	518659990	3.62
LI17M	Width	AX-94596631	1D	17533473	3.62
LI17M	TKW	AX-94596631	1D	17533473	4.18
LI18L	Length	AX-94766246	1D	418872302	3.54
LI17M	TKW	AX-94766246	1D	418872302	4.08
LI17M	TKW	AX-94865808	1D	17709714	3.13
LI17M	Width	AX-94865808	1D	17709714	3.43
ANN18M	Width	AX-94818538	2A	74116149	3.12
ANN18M	Length	AX-94818538	2A	74116149	3.91
LI18E	TKW	AX-95141980	2B	158603336	3.41
LI18E	Length	AX-95141980	2B	158603336	3.51
ANN18M	Length	AX-94610074	2D	81446063	3.53
ANN18M	Width	AX-94610074	2D	81446063	3.54
LI17L	TKW	AX-95107580	2D	189198358	3.35
LI17L	Width	AX-95107580	2D	189198358	3.36
LI17L	Width	AX-94986528	3B	795484711	3.10
LI17L	TKW	AX-94986528	3B	795484711	3.39
LI17L	TKW	AX-94469815	4A	616106716	3.14
LI17L	Width	AX-94469815	4A	616106716	3.40
ANN17E	TKW	AX-94426780	5A	597363315	3.26
LI17L	TKW	AX-94426780	5A	597363315	3.42
LI18E	Width	AX-94958973	5A	683270025	3.50
LI18E	TKW	AX-94958973	5A	683270025	4.21
LI17M	Length	AX-94476475	5B	714516404	3.28
LI17M	Width	AX-94476475	5B	714516404	4.24
LI17M	Width	AX-94890680	5B	714134727	3.01
LI17L	Width	AX-94890680	5B	714134727	4.10
LI17L	TKW	AX-94890680	5B	714134727	4.40
ANN18M	Width	AX-94394209	6A	1717618	3.17
ANN18M	TKW	AX-94394209	6A	1717618	5.36
ANN18M	Width	AX-94459169	6A	1719973	3.06
ANN18M	TKW	AX-94459169	6A	1719973	5.17
ANN18M	Width	AX-94484443	6B	54673673	3.06
LI17L	Width	AX-94484443	6B	54673673	3.31
LI17L	TKW	AX-94549328	6B	36319220	3.08
LI17L	Width	AX-94549328	6B	36319220	3.44
LI18E	TKW	AX-94439967	6D	23560832	3.01
LI18E	Length	AX-94439967	6D	23560832	3.49
LI17L	TKW	AX-95127531	6D	2385467	3.03
LI17L	Length	AX-95127531	6D	2385467	3.80
LI17L	TKW	AX-95132187	6D	488482849	3.44
LI17L	Width	AX-95132187	6D	488482849	3.52
LI18L	TKW	AX-95139913	6D	364760471	3.48
LI18L	Width	AX-95139913	6D	364760471	3.54
LI17L	TKW	AX-95157078	6D	488537244	3.63
LI17L	Width	AX-95157078	6D	488537244	3.75
LI18E	TKW	AX-95223861	7B	66518147	3.51
LI18E	Width	AX-95223861	7B	66518147	4.43
LI18L	TKW	AX-94435697	7D	56637758	3.21
LI18L	Width	AX-94435697	7D	56637758	3.21
LI18E	Width	AX-94463985	7D	551895334	3.05
ANN17E	Width	AX-94463985	7D	551895334	3.12

**Table 6:** Selected markers significantly correlated with climatic variables across environments for TKW, grain width, and length.

Marker	Chromosome	Position	Allelic Effect TKW	Significant Climatic Variable for TKW	Allelic Effect Width	Significant Climatic Variable for Width	Allelic Effect Length	Significant Climatic Variable for Length
AX-95224161	1B	518659990	−0.827	DiffDaylength (30 days after sowing)	−0.606	Average MaxTemperature (135 days after sowing)		
AX-95141980	2B	158603336	−0.773	DiffDaylength (150 days after sowing)			0.813	Average MinTemperature (30 days after sowing)
AX-95107580	2D	189198358	−0.669	ThermalInversion (90 days after sowing)	−0.774	Average MinTemperature (60 days after sowing)		
AX-94469815	4A	616106716	0.855	Daylength (15 days after sowing)	0.748	Average MaxTemperature (15 days after sowing)		
AX-94958973	5A	683270025	−0.706	Average MinTemperature (Heading Day)	0.667	Nightlength (15 days after sowing)		
AX-94890680	5B	714134727	0.767	DiffDaylength (30 days after sowing)	0.837	DiffDaylength (30 days after sowing)		
AX-94459169	6A	1719973	−0.876	Rainfall (Heading Day)	−0.750	Rainfall (Heading Day)		
AX-94549328	6B	36319220	−0.755	AccumDaylength (150 days after sowing)	−0.782	Average MinTemperature (135 days after sowing)		
AX-94439967	6D	23560832	0.739	AccumDaylength (135 days after sowing)			−0.725	ThermalInversion (30 days after sowing)
AX-95127531	6D	2385467	0.674	ThermalInversion (105 days after sowing)			−0.814	Average Temperature (Heading Day)
AX-95139913	6D	364760471	−0.830	ThermalInversion (90 days after sowing)	−0.870	Average MaxTemperature (60 days after sowing)		
AX-95223861	7B	66518147	0.798	Average MaxTemperature (90 days after sowing)	0.757	Average MinTemperature (90 days after sowing)		

## Data Availability

The data presented in this study are available in the article. The raw MS files are available on request from the corresponding author.

## Supplementary Materials

The following supporting information can be downloaded at: https://www.mdpi.com/article/10.3390/plants13111477/s1, Figure S1: Genome-wide association scan for Area, Circularity, Color, Perimeter, Roundness, and Volume of the WWAGI panel across environments; Table S1: Heritabilities of studied traits across environment; Table S2: Pearson correlations between all studied traits across environment; Table S3: Forward stepwise regression result for significant climatic variables effects and importance on each trait; Table S4: Multi-Environments Trials and winning genotypes of all traits under this study; Table S5: Significant marker associations that passes common threshold across environments; Table S6: Significant selected marker associated with climatic variables across environment for TKW, Width and Length; Table S7: Significant selected marker for TKW, Width and Length.
