# Supplementary material for: Dissection of the Genetic Basis of Genotype by Environment Interactions for Morphological Traits and Protein Content in Winter Wheat Panel Grown in Morocco and Spain
Source: Plants (Basel). 2024 May 27;13(11):1477. doi: 10.3390/plants13111477 (PMC11174427; doi:10.3390/plants13111477)
Supplement: Supplementary file 1 [file plants-13-01477-s001.zip › Figure S1 - Genome-wide association scan for Area, Circularity, Color, Perimeter, Roundness and Volume of the WWAGI panel across environment.pdf]

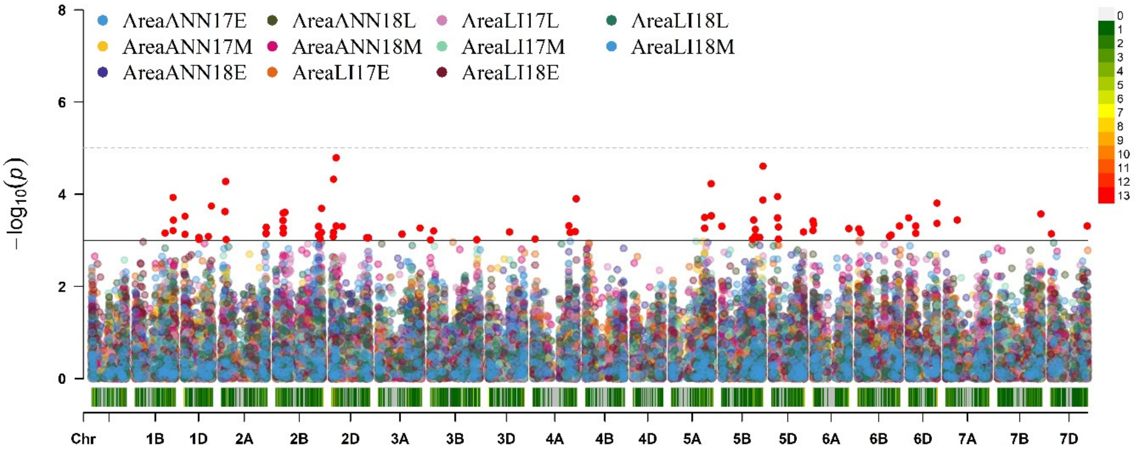
AreaANN17E
AreaANN17M
AreaANN18E
AreaANN18L
AreaANN18M
AreaLI17E
AreaLI17L
AreaLI17M
AreaLI18E
AreaLI18L
AreaLI18M
$-\log_{10}(p)$
Chr 1B 1D 2A 2B 2D 3A 3B 3D 4A 4B 4D 5A 5B 5D 6A 6B 6D 7A 7B 7D

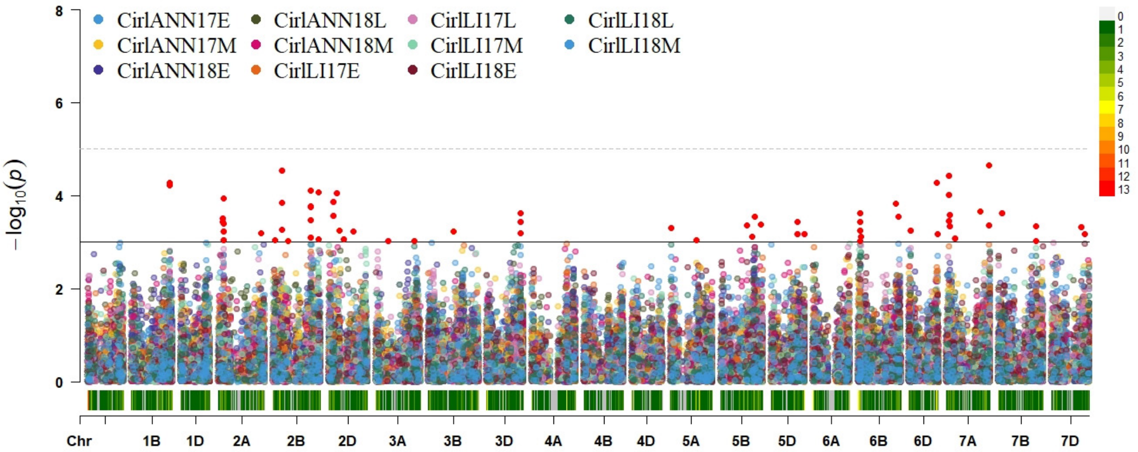
CirlANN17E
CirlANN17M
CirlANN18E
CirlANN18L
CirlANN18M
CirlLI17E
CirlLI17L
CirlLI17M
CirlLI18E
CirlLI18L
CirlLI18M
$-\log_{10}(p)$
Chr 1B 1D 2A 2B 2D 3A 3B 3D 4A 4B 4D 5A 5B 5D 6A 6B 6D 7A 7B 7D

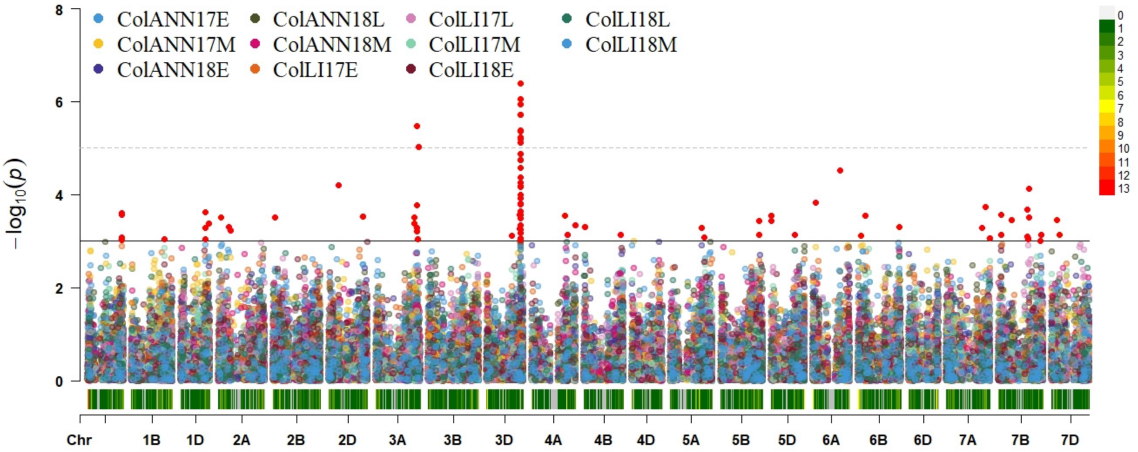
ColANN17E
ColANN17M
ColANN18E
ColANN18L
ColANN18M
ColLI17E
ColLI17L
ColLI17M
ColLI18E
ColLI18L
ColLI18M
$-\log_{10}(p)$
Chr 1B 1D 2A 2B 2D 3A 3B 3D 4A 4B 4D 5A 5B 5D 6A 6B 6D 7A 7B 7D

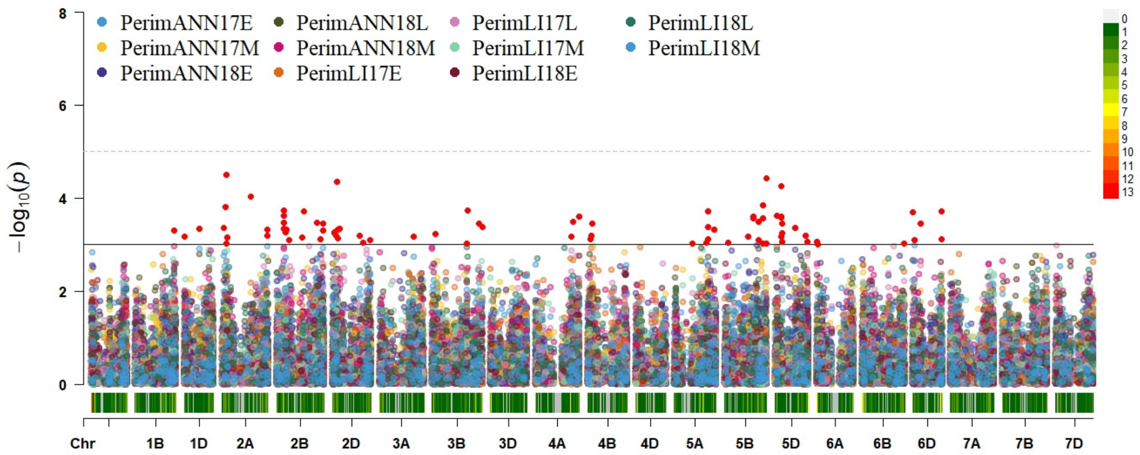
PerimANN17E
PerimANN18L
PerimLI17L
PerimLI18L
PerimANN17M
PerimANN18M
PerimLI17M
PerimLI18M
PerimANN18E
PerimLI17E
PerimLI18E
$-\log_{10}(p)$
Chr 1B 1D 2A 2B 2D 3A 3B 3D 4A 4B 4D 5A 5B 5D 6A 6B 6D 7A 7B 7D

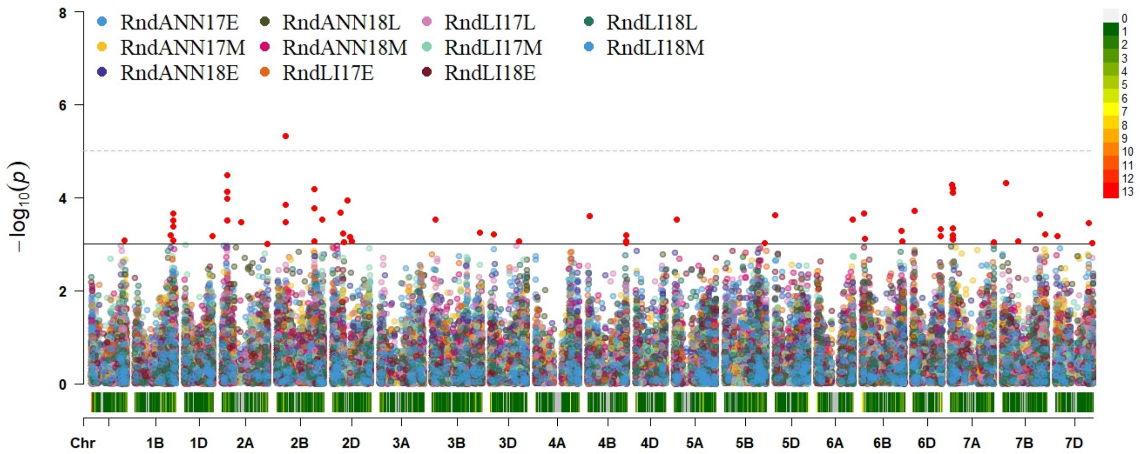
RndANN17E
RndANN18L
RndLI17L
RndLI18L
RndANN17M
RndANN18M
RndLI17M
RndLI18M
RndANN18E
RndLI17E
RndLI18E
$-\log_{10}(p)$
Chr 1B 1D 2A 2B 2D 3A 3B 3D 4A 4B 4D 5A 5B 5D 6A 6B 6D 7A 7B 7D

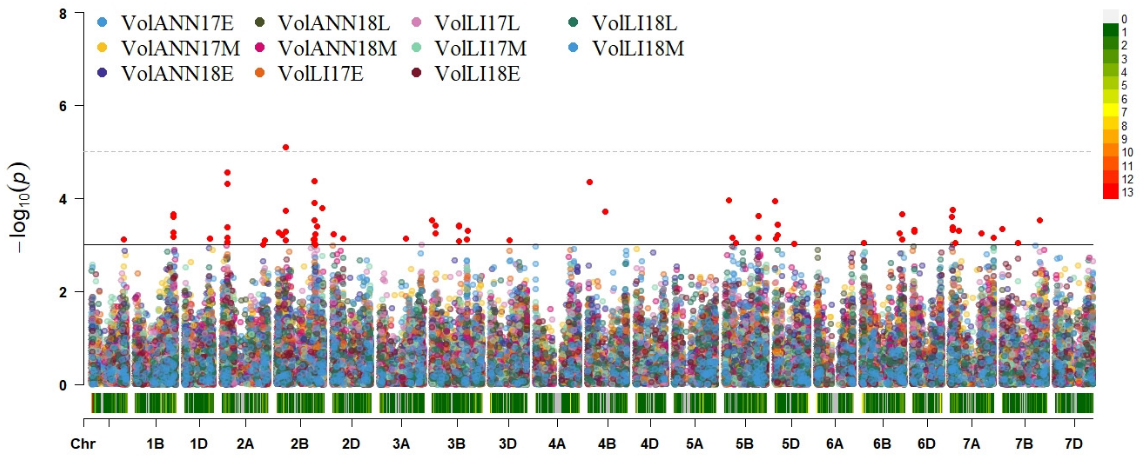
VolANN17E
VolANN18L
VolLI17L
VolLI18L
VolANN17M
VolANN18M
VolLI17M
VolLI18M
VolANN18E
VolLI17E
VolLI18E
$-\log_{10}(p)$
Chr 1B 1D 2A 2B 2D 3A 3B 3D 4A 4B 4D 5A 5B 5D 6A 6B 6D 7A 7B 7D

Figure S1: Genome-wide association scan for Area, Circularity, Color, Perimeter, Roundness and Volume of the WWAGI panel across environment. The plots show Manhattan plots with the significant SNPs. The chromosomes are shown on the X-axis and the genome-wide scan −log10 (P-values) are shown on the Y-axis using common threshold (-log10P>3) and Bonferroni correction (-log10P>5.2).
